# Supplementary material for: The genome sequence of Bocage’s wall lizard, Podarcis bocagei (Lopez-Seoane, 1885)
Source: Wellcome Open Res. 2025 May 19;10:246. [Version 1] doi: 10.12688/wellcomeopenres.24032.1 (PMC12260489; doi:10.12688/wellcomeopenres.24032.1)
Supplement: Peer review history

# Reviewer report: Reviewer response for version 1

**DOI:** 10.21956/wellcomeopenres.26514.r125439

**Published:** 2025-07-14

**Authors:** Sunandan Das

**Affiliations:** 1 University of Helsinki, Helsinki, Finland

In this report, authors report the reference quality genome for *Podarcis bocagei*. Using PacBio HiFi long read sequencing and Hi-C conformation capture sequencing, authors have generated 20 chromosome pseudomolecules. The sex chromosomes have also been identified. The data report is well written. The sequencing method used are generally the state-of-the-art for non-model wildlife. Assembly quality parameters are similar to values reported in contemporary non-model animal genome sequence reports/papers. The method is very detailed, including the in-silico steps, and should allow complete reproduction of the result with similar quality tissue sample. I regard this report to be a good contribution in *Podarcis *population and comparative genomics that should be approved.

Are sufficient details of methods and materials provided to allow replication by others?

Yes

Is the rationale for creating the dataset(s) clearly described?

Yes

Are the datasets clearly presented in a useable and accessible format?

Yes

Are the protocols appropriate and is the work technically sound?

Yes

Reviewer Expertise:

Systematics, Phylogenomics, Speciation Genomics, Herpetology

I confirm that I have read this submission and believe that I have an appropriate level of expertise to confirm that it is of an acceptable scientific standard.

# Reviewer report: Reviewer response for version 1

**DOI:** 10.21956/wellcomeopenres.26514.r124760

**Published:** 2025-06-13

**Authors:** Jessica Gomez-Garrido

**Affiliations:** 1 Centre Nacional d'Anàlisi Genòmica (CNAG), Barcelona, Barcelona, Spain

In this genome note, Feiner et al. present the genome assembly of *Podarcis bocagei*, a species of the genus podarcis that inabits the NortEast of the Iberian Peninsula. 28 different species have been described in the podarcis genus and some of these species also have available reference genomes. This new resource will allow comparative and evolutionary studies within the Podarcis genus and the Lacertidae family. 

 The sequencing and assembly strategies that the authors follow here are standard for generation of reference genome assemblies and give good results. Two chromosome-level haplotypes are reported here, with good percentage of chromosomal assignments. Both sex chromosomes have been identified and included in haplotype 1, although due to the repetitive nature of chromosome W, it has not been possible to fully reconstruct it. The mitogenome has also been assembled and included in the release. 

 Data has been correctly deposited and released in the INSDC databases. 

 Overall, I think this is a good new resource correctly described in this genome note. 

Are sufficient details of methods and materials provided to allow replication by others?

Yes

Is the rationale for creating the dataset(s) clearly described?

Yes

Are the datasets clearly presented in a useable and accessible format?

Yes

Are the protocols appropriate and is the work technically sound?

Yes

Reviewer Expertise:

Genome Assembly and Annotation

I confirm that I have read this submission and believe that I have an appropriate level of expertise to confirm that it is of an acceptable scientific standard.
